# Is IgG4-Related Disease a Cause of Xerostomia? A Cohort Study of 60 Patients

**DOI:** 10.1155/2012/303506

**Published:** 2012-10-16

**Authors:** M. Hermet, M. André, J. L. Kémény, G. Le Guenno, P. Déchelotte, G. Guettrot-Imbert, A. Tridon, I. Delèvaux, M. Soubrier, O. Aumaître

**Affiliations:** ^1^Department of Internal Medicine, Regional Competence Group on Adults' Auto immune and Rare systemic diseases, G. Montpied Hospital, University Hospital of Clermont-Ferrand, 63003 Clermont-Ferrand Cedex 1, France; ^2^Department of Pathology, G. Montpied Hospital, University Hospital of Clermont-Ferrand, 63003 Clermont-Ferrand Cedex 1, France; ^3^Department of Internal Medicine, Estaing Hospital, University Hospital of Clermont-Ferrand, 63003 Clermont-Ferrand Cedex 1, France; ^4^Department of Pathology, Estaing Hospital, University Hospital of Clermont-Ferrand, 63003 Clermont-Ferrand Cedex 1, France; ^5^Immunology Laboratory, Estaing Hospital, University Hospital of Clermont-Ferrand, 63003 Clermont-Ferrand Cedex 1, France; ^6^Department of Rheumatology, G. Montpied Hospital, University Hospital of Clermont-Ferrand, 63003 Clermont-Ferrand Cedex 1, France

## Abstract

*Objective*. Immunoglobulin-G4-(IgG4-) related disease (IgG4 RD) is a fibrosing process characterized by a significant infiltration of IgG4-secreting plasma cells. IgG4 RD can affect almost all organs including salivary glands. Whether IgG4 RD plays a role in the development of sicca syndrome and particularly dry mouth syndrome remains to be investigated. *Methods*. We conducted a monocentric cohort study for two years to search for IgG4 RD features in patients with dry mouth syndrome using immunostainings of labial salivary gland specimens with anti-IgG4 antibody. *Results*. Among 60 patients presenting with dry mouth syndrome who underwent labial salivary gland biopsy, 18 showed positive immunostaining with the anti-IgG4 antibody including 4 patients with typical systemic IgG4 RD. Five also fulfilled criteria for Sjögren's syndrome. *Conclusion*. These findings suggest that clinical forms of IgG4 RD salivary involvement without salivary swelling may occur. This salivary involvement is probably overlooked in everyday practice and could represent a mild form of IgG4 RD.

## 1. Introduction

The symptoms of dry mouth syndrome frequently cause patients to consult with physicians. More than 10% of the population reports symptoms linked to oral and/or ocular dryness. The causes of xerostomia are numerous including Kuttner's tumor (KT) and Mikulicz's disease (MD). Both are mainly characterized by enlargement increase in the volume of salivary glands, with submandibular glands involvement in KT and parotid glands in MD associated with lacrimal induration. Until 2005, MD and KT were classified as subtypes of Sjögren's syndrome (SS) [1] but the discovery of lymphoplasmacytic infiltrates rich in IgG4+ plasma cells in the salivary and lacrimal glands led to the reclassification of MD and KT as separate entities from SS. Nowadays, MD and KT are considered to be constituents of salivary involvement caused by immunoglobulin-G4-(IgG4-) related disease (IgG4 RD) [2]. KT is the third most frequent involvement of IgG4 RD (after bile ducts and lymphadenopathy involvement) in patients suffering from autoimmune pancreatitis (AIP) [3].

The concept of IgG4 RD was suggested in 2003 by Kamisawa et al. [4]. AIP, which was described much earlier, is part of this pathology and many publications are dedicated to this subject. IgG4 RD is a fibrosing process that can affect most organs and is characterized by a high number of plasmacytes that secrete IgG4. Essentially for epidemiological reasons, IgG4 RD has been the subject of Japanese-Korean studies although the first description was in French [5].

However, very little data are available in Western countries and the prevalence, characteristics of the salivary involvement of IgG4 RD, and its relationship to SS have yet to be defined.

The aim of this study was to determine if IgG4 RD features were present in French patients who had dry mouth syndrome whether or not they have SS. 

## 2. Patients and Methods

A cohort study was conducted retrospectively in a French university hospital for two years between January 2009 and December 2010.

 Thus, we reviewed all the medical charts of adult outpatients and patients who were hospitalized and had a labial salivary gland biopsy (LSGB) between January 2009 and December 2010, for any reason whatsoever. Patients with a context of sarcoidosis, amyloidosis, lymphoma, salivary lithiasis, drugs likely to induce a dry mouth syndrome, which had histories of cervical irradiation or systemic sclerosis, hepatitis C, or HIV infection were excluded. Then, immunostaining with anti-IgG and anti-IgG4 antibodies of the biopsy samples was performed for patients who presented with dry mouth syndrome according to the criteria in Box  1.

The LSGB samples were assessed in one of the two histopathology laboratories of this university hospital depending on which department the patient required. The LSGB samples were fixed in alcohol-formalin-acetic acid, dehydrated, embedded in paraffin, and sliced into six 5-micron-thick sections. An ultraView kit (VENTANA, Illkirch Cedex, France) was used to perform immunostaining with the sheep anti-IgG antibody IgG (Gamma-heavy chain) antibody-1 (Thermo Fisher Scientific, Cergy Pontoise, France) and the murine antihuman IgG4 antibody clone HP 6025 (the Binding Site, Saint Egrève Cedex, France). Diaminobenzidine was used as the reagent to visualize the antigenic sites. The immunostaining was performed in a single pathology laboratory. The IgG4/IgG tissue ratio was considered positive if the ratio was greater than or equal to 40%. Biopsies of other organs were examined by the same technique.

All included patients had positive Schirmer test and 47 of them were tested for autoantibodies to extractable nuclear antigen (ENAs). Those who were not tested for ENAs had a focus score equal to 1 or more on the LSGB. Measurement of saliva production had been performed by sugar test. A dry mouth syndrome was defined by not totally dissolved sugar after 3 minutes.

Using these data, the patients were divided into 4 subgroups according to the characteristics of their sicca syndrome: those who fulfilled SS criteria of the American-European Consensus Group [6] only (group A), those who fulfilled SS criteria and showed positive tissue immunostaining with the anti-IgG4 antibody (group B), and those who showed only positive tissue immunostaining (group C). A last group (group D) constituted a control group of patients who had sicca syndrome but no SS or IgG4 features (negative immunostaining).

When available, the following data were compared among the four groups: clinical criteria (age, sex, the presence of extrasalivary involvement, the notion of an atopic background, and salivary swelling), biological criteria (total IgG serum levels, IgG1, IgG2, IgG3, IgG4, and IgE serum levels, antinuclear antibodies (ANA) by IFI on HEP2 cells, ENAs including anti-SSA and anti-SSB on EliA Symphony Well Phadia (SAS, St Quentin Yvelines Cedex, France), rheumatoid factor (rf), and thyroid-stimulating hormone (TSH)), histopathological criteria (results of salivary tissue immunostaining with the anti-IgG4 antibody, the focus score, and the presence of interstitial fibrosis), and a therapeutic criterion (clinical response to steroid therapy).

We performed, with STATAv11 (StataCorp, College Station, TX), ANOVA test followed by a posthoc Tukey-Kramer test for continuous variables and Fisher's exact test followed by a Marascuilo procedure for categorical variables.

## 3. Results

Ninety-nine patients had an LSGB between January 2009 and December 2010. Among these 99 patients, 60 presented with subjective sicca syndrome according to the Vitali criteria [6]. 

The immunostaining could not be performed in 5 cases because the amount of the residual biopsy material was insufficient (*n* = 2) or visible lymphoplasmacytic infiltrates were absent (*n* = 3). 

The 55 remaining patients who had salivary tissue immunostaining with anti IgG4-antibodies were distributed among four groups depending on whether they met the diagnostic criteria for SS and/or have positive immunostaining with anti IgG4-antibodies on LSGB (*n* = 17) or on another tissue (*n* = 1) (as shown in Table 1).

### 3.1. Clinical Characteristics (Table 1)

The average age of the patients who had sicca syndrome was 61 ± 13 years. The average ages of each group were group A, 57 ± 13 years (25–76); group B, 66 ± 10 years (54–77); group C, 58 ± 7 years (47–73); group D, and 66 ± 14 years (38–89). There was no significant difference in the age between patients of groups A, B, C, and D. Of the patients who suffered from sicca syndrome, 30% presented with salivary involvement due to IgG4 RD with or without SS, 36.7% fulfilled only the diagnostic criteria of SS, and 33.3% did not fulfill the criteria for either pathology.

There were 49 women and 11 men with an average sex ratio (M/F) of 1/4.5. The highest proportion of women (1/5.5) was observed in group C.

An atopic background was observed in 18 patients including 8 in group A. Swelling of the main salivary glands (*n* = 4) occurred mainly in the patients from group C (*n* = 3), but the difference was not statistically significant. Lastly, extra-salivary involvement was observed in 46.6% of the cases (*n* = 28). 

Among the patients with extra-salivary involvement, some had arthritis (*n* = 13), central or peripheral nerve involvements (*n* = 5), haematological involvements (*n* = 3) including one patient with pernicious anaemia and vitiligo (*n* = 1), primary biliary cirrhosis (*n* = 2), fibrosing hepatitis (*n* = 1), interstitial pulmonary involvements (*n* = 2), thyroiditis (*n* = 1), dacryoadenitis (*n* = 1), skin involvements (*n* = 2 including livedo (*n* = 1), and urticaria (*n* = 1)), cervical lymphadenopathy (*n* = 1) and tubulointerstitial involvements of the kidneys (*n* = 2).

Two of the patients presented an extra-salivary involvement due to IgG4 RD that was proven histologically (liver *n* = 1, kidney *n* = 1).

### 3.2. Biological Characteristics

Due to the study design, the IgG4 serum level was measured in only 13 patients with a level greater than 1.35 g/L in 5 patients. Among these 5 patients, 4 showed characteristics of IgG4 salivary involvement only (*n* = 2) or associated with SS criteria (*n* = 2). The highest IgG4 serum level (26.1 g/L) was obtained in a patient with a systemic form of IgG4 RD. IgG4 serum levels tend to be higher in group B than in group A (*P* = 0.056). The serum levels of the other IgG subclasses (IgG1, IgG2, and IgG3) obtained in 7 patients were normal.

Autoimmune features (ANA and RF) were present in many patients, with ANA levels greater than or equal to 1/160 in 24 patients of the 54 who were tested and positive RF in 7 of the 28 patients. ANA levels (>1/160) were observed in 62% and 25% of tested patients in groups A and C, respectively, and in 60% of the patients in group B. The anti-ENA antibodies test was positive in 14 of 49 tested patients and most patients who tested positive belonged to group A (*n* = 11). ENAs positivity was significantly higher in group A than in group C (*P* < 0.0001) and tend to be more frequent in group A than in group D (*P* = 0.051). TSH measurement was performed in 26 patients and was elevated in 3, each of whom belonged to a different group (groups A, C, and D).

### 3.3. Histopathological Characteristics (Table 1)

A focus score equal to or greater than 1 was observed in 34 patients. Of these patients, 76% (*n* = 26) presented with SS criteria alone or associated with a positive IgG4 salivary immunostaining. A focus score equal to 1 or more was more frequently found in group A than in group C (*P* = 0.0058) and group D (*P* < 0.0001). A focus score equal to 1 or more was more frequent in group B than in group C (*P* = 0.0001) and group D (*P* < 0.0001). These expected results confirmed that a focus score equal to 1 or more was strongly associated with a diagnosis of SS, with or without features of IgG4 salivary involvement.

Interstitial fibrosis was observed in the LSGB samples in 30 of 60 patients, one-third of whom had IgG4 salivary involvement.

**Figure 1:**
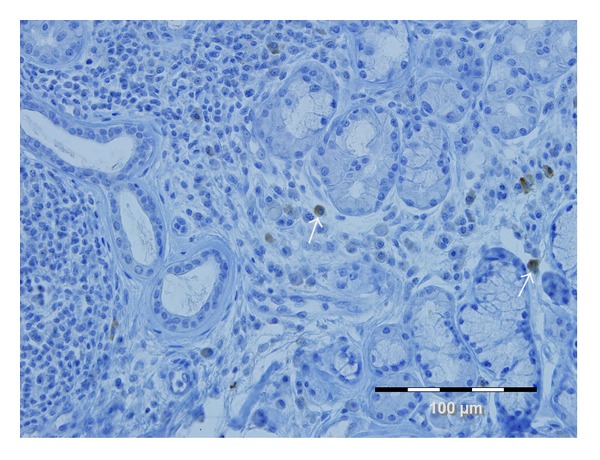
Infiltration of IgG-positive plasma cells in LSGB of patients with SS. Sections of LSGB were immunostained with hematoxylin and eosin (H&E) and anti-IgG antibodies. Diaminobenzidine (in brown on the picture showed by white arrows) was used as the reagent to visualize the antigenic sites.

**Figure 2:**
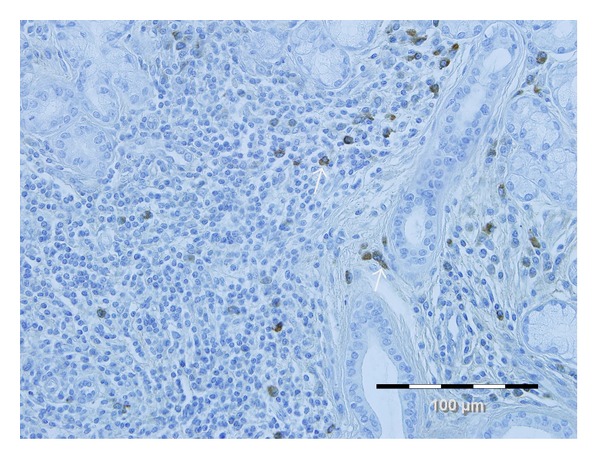
Infiltration of IgG4-positive plasma cells in LSGB of patients with SS. Sections of LSGB were immunostained with hematoxylin and eosin (H&E) and anti-IgG4 antibodies. Diaminobenzidine (in brown on the picture showed by white arrows) was used as the reagent to visualize the antigenic sites.

Immunostaining with anti-IgG4 antibodies showed positive results in 18 patients including 4 who belonged to group B and 13 patients who belonged to group C. The patients who had IgG4 salivary involvement features (groups B and C) showed positive immunostaining on LSGB more frequently than those from group A and D (group A versus B: *P* = 0.0002; group A versus C: *P* < 0.05; group D versus B: *P* = 0.0002; group D versus C *P* < 0.05) (Figures 1 and 2). One patient of the group B had a negative immunostaining with the anti-IgG4 antibody on LSGB but a positive one on liver biopsy. Positive immunostainings were defined by an IgG4/IgG positive plasma cells ratio >40%. Ratio and number of plasma cells per high-power field are available on Table 2.

### 3.4. Clinical Response to Steroid Therapy (Table 1)

Corticosteroids, given at the physician's discretion after the biopsy procedure, were used in 20 patients. No objective evaluation of the clinical response to steroid was performed. However, a subjective response in sicca syndrome was observed in 55% of cases (*n* = 11). Paradoxically, this improvement was not more marked in patients with stigmata of IgG4 salivary involvement. 

## 4. Discussion

Since MD and KT are recognised as part of IgG4 RD, few data are available about minor salivary gland involvement in this condition. Indeed, in the case of SS that represent a systemic cause of sicca syndrome, features of this disease are found on LSGB whether patients have or not parotid and submandibular glands enlargement, extrasalivary, or extra ocular symptoms. The occurrence of salivary swelling in the course of MD or KT is quasi-systematic and was as high as 100% of the patients from Geyer's study [7]. But recently, minor salivary glands involvements due to IgG4 RD have also been described [7, 8]. Although it concerns a limited number of cases, the current study showed the occurrence of salivary swelling was rare when IgG4 salivary involvement features were present but clearly more frequent than in the course of SS.

Tissue immunostainings with anti-IgG4 antibodies have been very useful in diagnosing IgG4 RD [9] even though if infiltrates rich in IgG4+ plasma cells are observed in other diseases such as rheumatoid arthritis, Crohn's disease, ulcerative colitis, and benign or malignant skin tumors [10]. However, an IgG4+/IgG+ plasma cells ratio greater than 40% in tissues appears to be a characteristic histological finding of IgG4 RD [10]. In our study, using this stricter criterion (international criterion), the sensitivity of IgG4-positive immunostaining for the diagnosis of IgG4 salivary involvement was 94.4% and the specificity was 100%.

Similarities in the phenotypes of both SS and IgG4 RD add to the confusion even if sicca syndrome has appeared to be less intense in patients with IgG4 salivary involvement than in those suffering from SS [1, 2, 11]. Many publications are dedicated to this topic and currently define MD as a symmetrical swelling of at least 2 glands among the lacrimal, parotid, and submandibular glands that persists for more than 3 months [1, 12]. The terminology for this salivary involvement is highly controversial, and at present, “sialadenitis linked to IgG4” is the most suitable expression. 

Three major studies have tried to address the clinical and biological differences between SS and MD or KT:

- in 2005, Yamamoto et al. [2] compared 7 MD patients with 14 SS patients and 12 patients who had a sicca syndrome without SS criteria,
- in 2010, Geyer et al. [7] compared 13 patients of Western origin who had a KT-type sialadenitis linked to IgG4 with patients who had chronic sialadenitis (*n* = 15), SS (*n* = 8), or lymphoepithelial sialadenitis, which is a benign pathological form of sialadenitis linked to SS in 40% of cases (*n* = 4),
- lastly, in 2009 and then in 2010, Masaki et al. compared 64 cases of MD in Japanese subjects with 31 cases of SS [11, 13].

The data from these studies showed that sialadenitis linked to IgG4 occurred more frequently in women but with a sex ratio (M/F) that was proportionally higher in men than in SS. Indeed, the sex ratio (M/F) in IgG4 sialadenitis in Asian patients appears to vary between 1/2 [11] and 1/3.6 [2] although it varies between 1/16.7 [11] and 1/20 [2] in SS. In European patients, the sex ratio (M/F) is 1/1.17 in IgG4 sialadenitis and 1/7 in SS [7]. In contrast to these results, our study found a sex ratio shifted toward women for IgG4 RD and toward men in SS.

The average age at onset of sialadenitis linked to IgG4 varies between 61 [7] and 66.7 years [2]. The age of onset of SS is usually lower (at 56 years) [7]. The patients from our study with SS only were significantly younger than those from the control group but we did not observe an age difference between patients with SS and those with IgG4 salivary involvement features.

One group of our patients, even though small, fulfilled the SS criteria and showed IgG4-positive immunostaining. These findings suggest an eventual coexistence of SS and IgG4 salivary involvement features in patients with sicca syndrome. However, a focus score ≥ 1 was found in our patients with the expected frequency reported in the literature [14]. This observation questions the current diagnostic criteria of IgG4 salivary involvement that require exclusion of SS before IgG4 RD can be diagnosed [11] and reciprocally.

The relationships between IgG4 and allergic disorders were described in 1974, fuelling the debate on whether the origin of IgG4 RD was autoimmune or allergic [15]. In our study, we did not show a higher prevalence of atopic manifestations in patients with IgG4 salivary involvement. However, despite the very low number of patients who had an IgE test, we found significantly higher IgE levels in the patients with IgG4 salivary involvement features than in those with SS. On the other hand, allergic disorders have also been found more frequent in Sjögren's syndrome than in osteoarthritis patients [16].

A serum IgG4 level greater than 1.35 g/L was detected significantly more frequently in the group with SS associated with IgG4 salivary involvement features than in the group with SS alone. Regardless of the affected organ, the IgG4 serum level is often significantly higher in IgG4 RD than in other causes [17]. These data also apply to MD [2, 11]. However, isolated elevation of IgG4 serum levels without a clinical context or pathological confirmation should not lead to a hasty diagnosis of IgG4 RD [18]. The Asian consensus for IgG4 RD retains a level greater than 1.35 g/L [19].

Autoimmune biological features are usually observed in IgG4 RD patients. In salivary involvement, the presence of ANA and positive RF was frequent. The presence of ANA occurred in approximately 28% of cases in Yamamoto's study [2] and 25% in our patients with IgG4 salivary involvement features. Hypocomplementemia occurred more frequently in MD than in SS [3]. However, anti-SSA and anti-SSB antibodies were very typical of SS in our study, a finding in accordance with the literature data [2, 3, 11].

From a pathological point of view, IgG4 RD is characterized by the association of a lymphoplasmacytic infiltrate rich in IgG4+ plasmacytes, a fibrosis, and in situ thrombosis in variable proportions [9]. Fibrosis is rare in SS and clearly more frequent in IgG4 salivary involvement [9]. The results of our study did not show noteworthy differences in the presence of fibrosis among the four groups. The location of the fibrosis (peri- or intralobular in SS and often interlobular in IgG4 salivary involvement) can help the pathological diagnosis. The presence of in situ thrombosis could not be evaluated in our patients given the type of histological sample that was used [20].

From a therapeutic point of view, the steroid responsiveness of IgG4 salivary involvement is well known [12]. MD or KT types of salivary involvements follow the same rule [1–3, 11]. Yamamoto et al. showed a significant improvement in salivary flow with corticoids treatment in patients suffering from MD, which was contrary to the finding in patients with SS [2]. According to Reksten et al.'s study [21], steroid therapy has a positive effect on the extraglandular manifestations of primary Sjögren's syndrome. Our data does not allow us to conclude on a stronger steroid responsiveness of IgG4 salivary involvement. 

To the best of our knowledge, our study included the largest cohort of patients of Western origin with IgG4 salivary involvement features. The findings highlight the possible association between IgG4 salivary involvement features and SS, which leads us to believe that an overlap of these two pathologies exists. Furthermore, the clinical characteristics of the patients in our study show probable phenotypic differences in IgG4 salivary involvement between the Asian and Western patients. 

There are some limitations in our study. First, the control group comprises patients that are “nonhealthy” because of sicca syndrome and the 4 groups are not matched. Second, as in all retrospective studies, our clinical and paraclinical data are not exhaustive. 

## 5. Conclusion

Of the 60 French patients in our series who suffered from sicca syndrome, 30% showed stigmata of IgG4-related sialadenitis with or without SS. The results of our study suggest that mild clinical forms of IgG4 salivary involvement with a low prevalence of salivary swelling could exist. This disease is probably underestimated in everyday practice and might constitute a nonexceptional cause of sicca syndrome. Some degree of overlap between SS and sialadenitis linked to IgG4 could also occur to a small extent.

Our findings suggest that, in cases of sicca syndrome that do not match SS criteria, IgG4 salivary involvement should be considered and immunostaining with the anti-IgG4 antibody of a LSGB sample may be useful for diagnosis. To assess the efficiency of corticotherapy on dryness symptoms of patients with IgG4 salivary involvement would require a larger study, but this therapeutic option is interesting.

## Figures and Tables

**Box 1:**
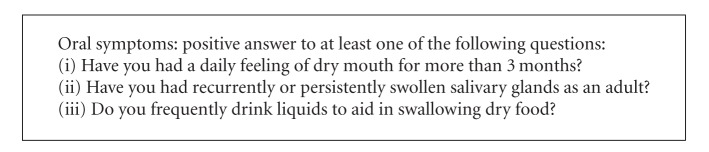
Characteristics of subjective dry mouth syndrome according to Vitali et al. [6].

**Table 1:** Clinical, histopathological, and therapeutic data.

Group	A	B	C	D
SS criteria	Yes	Yes	No	No
Positive immunostaining with anti IgG4 antibodies	No	Yes	Yes	No
No. of patients (total *n* = 60)	22	5	13	20
Age	57 ± 13	66 ± 10	58 ± 7	66 ± 14
Sex ratio (M : F)	1 : 4.5	1 : 4	1 : 5.5	1 : 4
Atopic background	8	1	2	7
Salivary glands swellings	0	0	3	1
Extra-salivary involvements	11	4	6	7
Focus score ≥ 1	20	5	6	3
IgG4+ immunostaining on LSGB	0	4	13	0
Fibrosis	13	3	7	7
Corticotherapy	3	2	7	8
Response to steroids	1	0	4	6

**Table 2:** Results of immunostaining with anti-IgG and anti-IgG4 antibodies. Ratio > 40% means that plasma cells have not been counted (when >50/HPF).

Patient	1	2	3	4	5	6	7	8	9	10	11	12	13	14	15	16	17	18
Ratio IgG4+/IgG+	2/2	3/6	3/6	2/1	41/46	5/12	2/2	1/1	12/7	3/6	5/12	10/7	4/5	2/2	>40%	>40%	>40%	>40%
